# Learning how: A “mindless” procedure alone gives rise to contextual cueing—A weakly supervised connectionist model of statistical context learning in visual search

**DOI:** 10.3758/s13423-026-02999-0

**Published:** 2026-09-30

**Authors:** Werner Seitz, Artyom Zinchenko, Hermann J. Müller, Thomas Geyer

**Affiliations:** 1 Department Psychologie, Lehrstuhl Für Allgemeine Und Experimentelle Psychologie, Ludwig-Maximilians-Universität München, Leopoldstraße 13, 80802 Munich, Germany; 2 Graduate School of Systemic Neurosciences, Ludwig-Maximilians-Universität München, Munich, Germany; 3 Munich Center for Neurosciences – Brain & Mind, Ludwig-Maximilians-Universität München, Munich, Germany; 4 NICUM – NeuroImaging Core Unit Munich, Ludwig-Maximilians-Universität München, Munich, Germany

**Keywords:** Visual search, Selective attention, Statistical learning, Contextual cueing, Hebbian learning, Computational modeling

## Abstract

Because our environment is not random, it is beneficial to assimilate the statistics of sensory impressions and improve performance, such as visual search for a target object in a cluttered array of nontarget objects (contextual cueing [CC] effect). Computational models of CC have so far focused on predicting the target location from a particular configuration of nontarget items. This contrasts with recent findings according to which display repetitions train human participants’ general procedures for the search task. Here, we test the latter idea by employing a connectionist model of visual search that exclusively learns a search procedure without acquiring any individual display-layout information. We show that an instance of a “learning how” mechanism not only proposes a viable alternative account to existing “learning that” mechanisms, but also generates more plausible key behavioral metrics and exhibits a central bias as an emergent phenomenon of learning-induced plasticity. These findings have implications for models of visual search and artificial intelligence: Learning a procedure from leveraging a task’s structure alone can mimic the effects of top-down modulation of attention while also reducing the need for supervision in learning, thereby making computational models that leverage procedural learning behaviorally more plausible and easier to train.

## Introduction

Detecting, and responding to, specific objects in a cluttered sensory world is one of the most prevalent tasks of the human visual system (Wolfe, 1998; Wolfe & Horowitz, 2017). Since environments are typically stable, it is beneficial to extract information that is predictive of relevant “target” objects and use this information to facilitate search on later occasions. For example, looking for a certain item in a shop for the first time is driven by knowledge about the item’s featural (size, color, etc.) properties. In subsequent searches for the same item in the same shop, another source of information becomes available: knowledge about the location of the target item on a certain shelf in the presence of other distractor (i.e., nontarget) items that, while not directly relevant for the task at hand, nevertheless provide a helpful context for finding the searched-for item.

An experimental paradigm that mimics this situation in the laboratory, originally introduced by Chun and Jiang (1998), is visual search for a target item placed at a stable position within a constant (i.e., repeated) spatial arrangement of distractor items. The basic task requires participants to search through artificial arrays of letter-type stimuli, finding and responding to the orientation of a T-shaped target (rotated by either 90° or 270°) among (orthogonally oriented) L-shaped distractor items. Critically, search performance for repeated distractor-target arrangements, in which the distractor layout, or “context,” is predictive of the target location, is compared against nonrepeated (“baseline”) arrangements, in which distractor locations are arranged anew on each trial. The target locations themselves are fixed in both repeated and nonrepeated displays, controlling for absolute target-position learning (cf. Geng & Behrmann, 2005). This laboratory task thus allows the isolated study of spatial context learning, unconfounded by semantic and syntactic properties of the searched scenes (for evidence that such properties matter, see Võ & Wolfe, 2013). Chun and Jiang (1998, 2003) found the search reaction times (RTs) to decrease with increasing practice on the task not only for repeated but also for nonrepeated display arrangements, reflecting procedural learning of how to best perform the task, akin to the acquisition of a (search) skill (Fitts, 1954; Fitts & Posner, 1967). Critically, the practice-related improvement turned out larger for repeated versus nonrepeated displays, which has been attributed to the beneficial effect of repeated distractor contexts for finding the target. Chun and Jiang (1998) proposed that repeatedly encountered context–target spatial relations are stored in spatial long-term (LT) memory. Upon reencountering a repeated display, the appropriate memory is retrieved and guides, or “cues,” search to the target location, giving rise to the “contextual-cueing” (CC) effect.

Central to accounts of CC is the more efficient allocation of focal attention to the target item in repeated displays (Chun & Jiang, 1998; for reviews, see Goujon et al., 2015; Sisk et al., 2019). In support of this, oculomotor investigations of CC have shown that detecting the target in repeated displays requires fewer eye movements (i.e., a reduced number of fixations, NF, where, in a fixation, covert and overt attention are assumed to be aligned) compared with the baseline of nonrepeated displays (Peterson & Kramer, 2001; Tseng & Li, 2004). Relatedly, oculomotor scan paths (i.e., the total distance traversed by sequential eye movements) are also shorter for repeated displays (e.g., Brockmole & Henderson, 2006; Manginelli & Pollmann, 2009).

Dominant theoretical accounts of CC attribute this pattern of effects to the build-up of contextual memories that associate the location of the target with the distractor layout within specific repeated display arrangements (Beesley et al., 2015; Chun & Jiang, 1998; Tseng & Li, 2004). In terms of Ryle (1946), such theories of CC may be considered instances of “knowing that” (i.e., what is acquired is some kind of [search-guiding] proposition that, given a certain distractor layout, the target is to be found at location [*x,y*]). Complementary to this would be accounts of “knowing how”: The capability to optimally perform specific tasks or actions (Ryle, 1946). Arguably, the CC paradigm is particularly well suited to investigating the latter type of account, which considers the refinement of the requisite (i.e., overall optimal) search procedure to be driven more strongly by repeated than by nonrepeated displays.^1^ In line with this notion, recent analyses of the oculomotor scan paths observers traverse to find the target show that procedural learning of how to the search set (or “world”) of displays encountered is an important determinant of the CC effect (Seitz et al., 2023, 2024): while scan-path similarity increases generally across all types of displays, with training, critically, it increases more for repeated than for nonrepeated displays. This supports a “knowing-how” account of CC, according to which statistical learning optimizes the processes by which the search task is accomplished, with repeatedly encountered contexts simply accruing a greater weight in tuning these processes than nonrepeated contexts, which, by definition, are searched only once.

Given this evidence, the motivation of the present study was to test computationally whether procedural learning of how to optimally search a provided set of displays can give rise to a CC effect in the absence of any display-specific learning (i.e., without associating a specific distractor arrangement with a specific target location). Along with this, we set out to test whether an only weakly supervised model—leveraging the implicit structure of the task, rather than relying on an explicit teaching signal (i.e., a label in form of the target location)—could still exhibit adaptive behavior. If so, this would not only elucidate a thus far underrepresented form of learning in visual search, but, beyond that, have implications for AI in that it holds the possibility of designing/collating new datasets with a smaller burden on explicit annotation.

### Previous models of CC

Based on the findings of faster response times (RTs) and fewer fixations required for detecting targets in repeated contexts, there have been several attempts to model the cueing, or guidance, of attention to learnt target locations by acquired long-term contextual-memory representations. Brady and Chun (2007) proposed a two-layer feedforward network that learned to associate a specific context with a specific target position. The input layer consisted of an 8 × 6 (location) matrix, with “occupied” matrix cells encoding the presence of one out of a total of 12 display items at the respective location. Each display arrangement then activated (via a bottom-up activation term) spatially corresponding neurons in an output layer that was considered to represent a priority map (cf. Fecteau & Munoz, 2006) for the deployment of attention and eye movements. The input and the output layer were connected by weights that were learned to associate a target position with a given display arrangement according to the delta rule, with a spatial weighting: weight updating was largest for the target location and (exponentially) decaying for distractors with increasing spatial distance from the target location. The network’s performance was measured in terms of the number of activated output-layer units that had to be checked in decreasing order prior to arriving at the unit representing the target location. This model was well able to generate a CC effect, in terms of a reduction in the number of fixations required to find the target in repeated display layouts. An augmented version of this basic two-layer network was proposed by Beesley et al. (2015) to account for the observation of facilitated RTs even when the target location is allowed to vary relative to an invariant distractor layout (though, in this case, the facilitation is smaller than the standard CC effect with fixed target locations; e.g., Kunar & Wolfe, 2011; Vadillo et al., 2021). To accommodate this finding, Beesley et al.’s model learns an additional set of auto-associative weights between distractors within the input layer, reinforcing the distractor configuration.

Thus, in these modeling attempts, associations are formed (i.e., connections are strengthened) between the spatial configuration of items in the input layer and the location of the searched-for target in the attentional-priority output layer in a supervised manner (for an overview, see Dayan & Abbott, 2005); additionally, associations may be acquired among repeatedly placed distractor items within the input layer using unsupervised learning. These models correspond to the notion of “learning that” (see above), mainly involving display-specific learning, as we will outline in the discussion.

## Present work

**Fig. 1:**
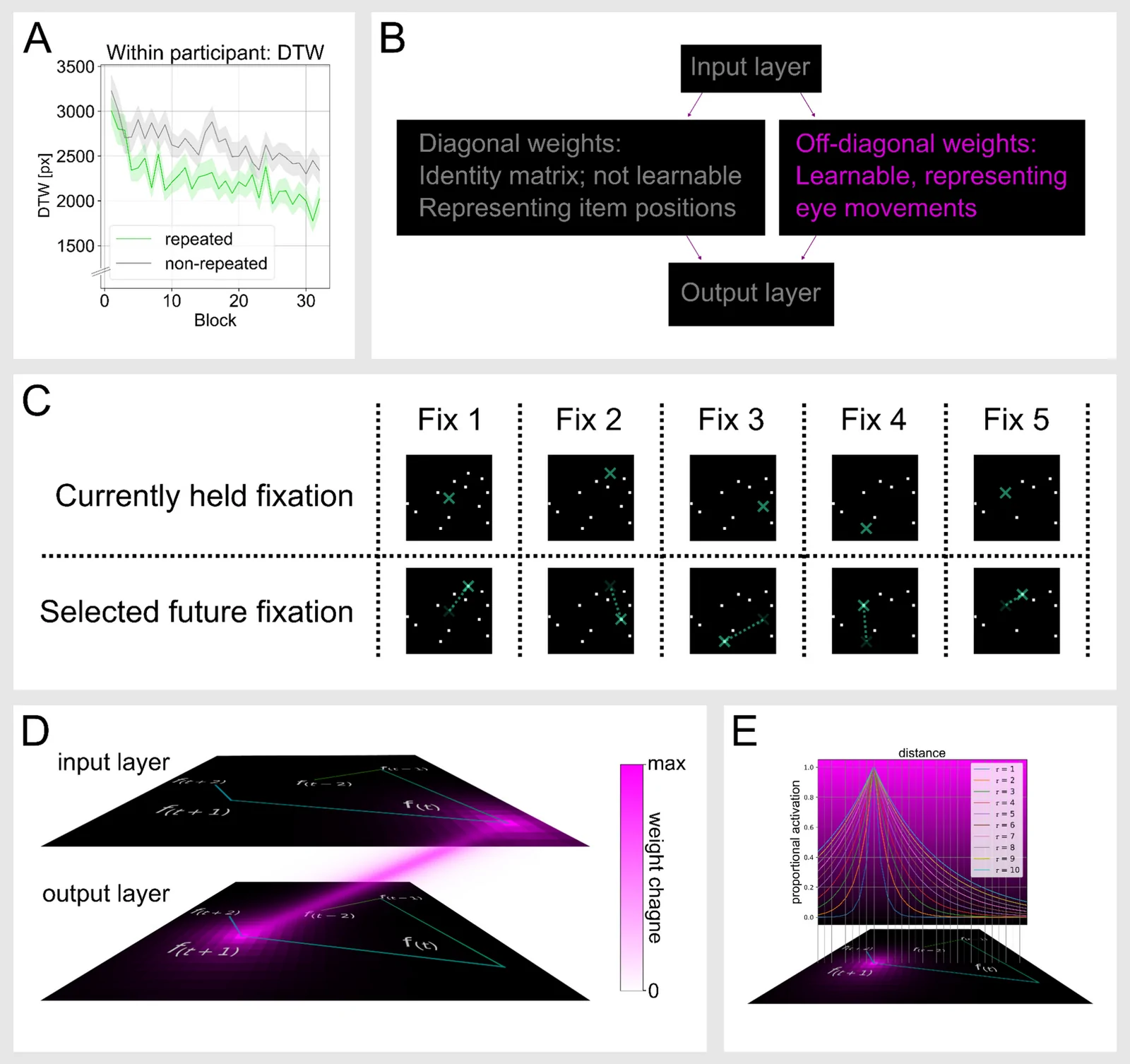
**A** Results from human participants (from Seitz et al., 2023) who performed visual search of repeated and nonrepeated target-distractor layouts over 32 trial blocks; this led to a practice-related (i.e., block-related) increase in scan-path similarity (measured by Dynamic Time Warping [DTW]; see, e.g., Fahimi & Bruce, 2021) with increasing time-on-task (learning), with a greater increase for repeated versus nonrepeated displays. **B** Architecture of the network examined in the present study, which consisted of two layers that are connected by two sets of (fixed and, respectively, learnable) weights. **C** Outline of the overall dynamics of the model: The initial fixation is set at the display center and a particular item is selected as the next fixation location, which is then “removed” from the input prior to determining a new fixation location. This iterative selection-and-rejection process is terminated upon a fixation falling on the target. **D** Visualization of the amount of weight change resulting from Hebbian learning between laterally activated neurons around the current fixation (in the input layer) and those of the selected next fixation (output layer). **E** Illustration of the decreasing amount of lateral activation of a given neuron as a function of its distance from the fixation location. Smaller values of τ (the parameter controlling the spatial decay) yield a faster decrease in activation. (Color figure online)

While associative learning (Goujon et al., 2015) presents a plausible mechanism for generating contextual facilitation as a form of statistical learning, the levels at which statistical regularities may actually be leveraged have not yet been comprehensively investigated, at least not in terms of computationally explicit models. Recent empirical work (Seitz et al., 2023, 2024) has demonstrated that contextual facilitation—rather than being simply attributable to the acquisition of specific, repeatedly encountered distractor–target relations (display-specific learning)—may arise from procedural, oculomotor–scan-path learning adapted to the set of encountered display layouts at large (display-generic learning; i.e., the honing of a general search “skill” in terms of Fitts & Posner, 1967). Of note, display-specific learning as such could account only for the acquisition of a search advantage for repeated over nonrepeated displays. In contrast, acquiring a generic procedural strategy to optimally scan the entire set of encountered displays (including both repeated and nonrepeated layouts) can provide a parsimonious account of both contextual facilitation (i.e., the search advantage for repeated vs. nonrepeated displays) and the general improvement in performance across time-on-task, which is evident with both repeated and nonrepeated displays: while scanning is optimized for all types of display (accounting for the general improvement in performance), repeated displays are encountered multiple times and thus shape the scanning strategy more than nonrepeated displays that are encountered only once (for detailed analyses and discussion, see Seitz et al., 2023). As a result, the similarity of the oculomotor scan paths becomes higher between pairs of (differently composed) repeated displays compared with pairs of (different) nonrepeated displays (see Fig. 1A). This proposal of display-generic (rather than display-specific) learning is in line with Lashley’s (1951) account of sequential behavior, according to which motor actions are not planned de novo each time, but instead previously executed movement patterns are modified or updated to fit the demands of the task at hand (see also Rosenbaum et al., 2007).

Based on this idea and the converging evidence from measures of scan-path similarity (Seitz et al., 2023, 2024), we implemented a computational model which, by virtue of its architectural constraints, cannot in principle form any contextual associations between a display arrangement and a target location (output layer); rather, it can only learn fixation sequences based on previously executed saccades, in accordance with theories of procedural learning (Lashely, 1951). Beyond this theoretical motivation, choosing this simple procedural learning rule was also underpinned by a pragmatic, evolutionary consideration: Executing stereotypical eye movements driven by locations of relevance in the current input, as well as indirectly by those of previously “scrutinized” displays, would, by itself, provide a fairly economical way of optimizing where and in which sequence fixations should be placed. Given such a fixational sampling policy, the fixations will be tuned to general characteristics of relevance in the set of displays encountered and thus be likely to capture the salient, evolutionarily informative aspects of the input. Hence, the assumptions underlying the present scheme differ fundamentally from those of the previous models, which learn target locations as a function of context: the models inspect likely candidate positions guided by initially erroneous memory signals, that, across repeated searches, become gradually biased towards the target location. In contrast, the model investigated here does not acquire any associations between an item configuration and the target location predicted by that configuration; instead, it optimizes its behavior as an emergent property of reinforcing previously executed oculomotor patterns.

Thus, the present modeling attempt present a fundamental departure from previous schemes: Employing a computational model which, by design, can only learn a scanning procedure and no spatial-layout information at all allows us to examine whether contextual facilitation can arise merely as byproduct of an acquired, essentially “mindless” generic search procedure. Here, “mindless” is used in a strictly operational sense to denote that learning is not driven by explicit representations of task-relevant variables such as target identity, target location, or stimulus–response mappings. Instead, learning is based solely on local, sequential transitions between fixations, without any mechanism that encodes or retrieves display-specific configurations or goal-related templates. Importantly, this does not imply randomness or lack of structure; rather, structured behavior emerges from simple, local learning rules operating over fixation-to-fixation transitions. If so, the model results would demonstrate that to account for contextual facilitation, it is not necessary to assume the build-up of higher-level contextual-memory “templates” in spatial long-term memory that are activated by the presentation of a specific repeated display and then top-down bias search towards the target location pointed to by the distractor configuration. Of note, our model incorporates the notion of CC being a mere byproduct of associative learning (cf. Goujon et al., 2015), though with associative learning in the model occurring simply between successive fixations, rather than extracted regularities in the item arrangement.

In terms of behavioral indices of CC, following previous studies (Beesley et al., 2015; Brady & Chun, 2007), we focused on the key measure of the number of fixations required by the model to detect the target. Further, for a more comprehensive validation of our computational model against the evidence of CC being driven by procedural learning (Seitz et al., 2023), we additionally examined the model’s search behavior in terms of scan-path-similarity measures (i.e., the homogeneity of the scan paths within “participants” and between displays). Although these two measures are related, they index different aspects of learning-related changes in visual search behavior. The number of fixations provides an index of overall search efficiency, reflecting how quickly the target is located. In contrast, scan-path similarity captures the degree to which learning leads to convergence in the structure of oculomotor behavior (i.e., whether repeated encounters with displays result in increasingly stereotyped sequences of eye movements). Thus, while the two measures are expected to correlate, they are not redundant. If procedural learning alone suffices to produce CC, we expected the model’s behavior to replicate previous findings diagnostic of procedural learning, in particular: a reduced number of fixations as well as a higher similarity of the scan paths in later versus earlier blocks of learning, especially for repeated versus nonrepeated displays.

## Methods

The model was implemented in PyTorch (Paszke et al., 2019), allowing for CUDA (Release: 10.2.89; NVIDDIA, https://developer.nvidia.com/cuda-toolkit) optimized code, and was trained on an NVIDIA RTX A4000 GPU. Like previous models of CC, the current model has two layers, an input layer and an output layer, connected via a set of weights, with each element in the input and output layer corresponding to a possible item location—similar to Brady and Chun’s (2007) model. Thus, architecturally, the model is consistent with the prior literature, and the potential contributions of the modeling outcome relate to the search and learning mechanisms involved in CC (for further details, see below). The architecture of the model is illustrated in Fig. 1B.

### Search stimuli

As in previous models (see, e.g., Brady & Chun, 2007), the input of the model consisted of a matrix in which positions that were occupied (either by a target or a distractor) were encoded with the value 1 for that element, while unoccupied elements were set to 0. Since the model was meant not to learn context-target relations, but to examine the learning of fixation sequences, the display matrix was increased to 31×31 locations, allowing for more versatile scanpaths. Each item was encoded by setting its corresponding location to 1, regardless of whether it was a distractor or a target. There were 12 unique (i.e., fixed) target positions for repeated and 12 different positions for nonrepeated displays; target positions were counterbalanced across the four display quadrants, though any location was equally probable. Otherwise, the likelihood of an item appearing anywhere in the display followed a uniform distribution, with the constraint that no two items could share the same position.

For the baseline task, displays were created for 32 trial blocks. In each block, the 12 repeated displays were presented as well as 12 nonrepeated displays—the latter with a constant target position but otherwise variable distractor positions, adhering to prior studies (Chun & Jiang, 1998; Peterson & Kramer, 2001).

In order to assess the model’s generalization regarding theoretical predictions from the literature, two variants of the baseline task were assessed by means of presenting different stimuli. The first variant addressed the locality of learning, as the literature indicates that distractor-context learning is more effective in the spatial vicinity of the target than further away from it (see Olson & Chun, 2002; Brady & Chun, 2007). Accordingly, in baseline-task variant 1, either the distractor items in the target quadrant or those in the opposite quadrant were placed at novel (i.e., nonpredictable) positions in the last trial block, in order to assess whether there was a search cost (in terms of an increase in the number of fixations relative to the previous block) whether this cost was higher if the items of the target (vs. a nontarget) quadrant were placed at new positions.

The second variant assessed target relocation—that is, the elimination (and subsequently slow recovery) of the CC effect after the target is consistently shifted to a new position and replaced by a distractor at the old position (Manginelli & Pollmann, 2009; note that a CC effect reemerges). Accordingly, after 32 blocks of learning, a relocation phase for another four blocks was added in which the target was repositioned in the opposite (to the original) quadrant (as in Seitz et al., 2024; Zinchenko et al., 2020), in order to assess whether a search cost could be observed for the block or the “epoch” after target relocation, where an epoch summarizes performance across several blocks (the summary measure employed in most relocation studies; e.g., Geyer et al., 2024; Zinchenko et al., 2020).

### Search mechanism

Previous models (Beesley et al., 2015; Brady & Chun, 2007) did not implement a specific search “procedure”: rather, for a given (presented) input arrangement, the model yielded an activation value for each occupied location—the underlying (implicit) assumption being that the model then checks those locations, starting at that with the highest activation, in decreasing order until arriving at the target location (however, no learning occurred during this theoretical search, and only the target location was learned with respect to the entire presented display, regardless of the hypothetical sequence of fixations). In contrast, here we implemented a dedicated search procedure: The model explicitly computes an oculomotor scan path (i.e., a sequence of fixation coordinates), starting at the display center as the initial location, and then successively sampling locations one after the other until the target is located (similar to the model of Koch & Ullman, 1984, or the Target Acquisition Model of Zelinsky, 2008).

At each “current” fixation, the model selects a location for the next fixation. This location is either the output neuron (item location) exhibiting the highest activation, or, if there are several output neurons with equal activation (i.e., before learning; for details, see below), it randomly selects one of those. If the selected item location does not contain the target, the selected item is removed from the input layer (as a result of which it is no longer represented on the output layer and, so, its location can no longer be selected for fixation in future steps)—implementing an “inhibition-of-return” mechanism (e.g., Klein & MacInnes, 1999; Koch & Ullman, 1984; Posner & Cohen, 1984). From this (“rejected”) location, the location of the next fixation is determined, and scanning is terminated once the model selects the target location. Note that, as the model favors high activation values, search is driven by occupied item locations (i.e., nonzero elements in the input), though nonoccupied locations in the output layer receive weak activation from lateral activations (for details, see “Learning” section). An illustration of this search procedure for an example display is provided in Fig. 1C. After selecting the target, the fixation location is reset to the display center, with a new display presented as input.

In short, our model learns to associate a current fixation location in the input layer with the selected-to-be-next fixation location in the output layer using Hebbian learning. During visual scanning of the (output layer) environment, fixations can either land at (exact) distractor or target locations, and in case of the latter—target-fixation—event, search will be terminated. Consequently, weights are updated for a specific item (i.e., distractor and target) locations. However, the weight modulation also spreads (a lesser, spatially scaled, extent) to locations in the vicinity of fixated items. Critically though, while such nearby locations may not necessarily contain a distractor or target item in the current search display, they may be occupied by search items in other display arrays. Accordingly, output units learned from a given, searched display would come to modulate the input–output relations in other displays. Thus, by employing Hebbian learning with lateral activations, we can implement the concept of procedural learning of, across displays, shared fixational policies that generalize over space, eventually leading to search facilitation with increasing time on task.

### Learning

Architecturally, the model is a feedforward network with two layers, connected by a weight matrix. While full connectivity between each neuron in the input layer and every neuron in the output layer is possible, initially only the diagonal elements are nonzero. These diagonal weights connect spatially corresponding positions in the input and output layers and maintain a fixed value (i.e., they do not change as a result of learning). Essentially, they provide a “bottom up” signal that represents the item locations in the output layer (with equal “priority”), similar to the bottom-up weights in Brady and Chun (2007). Thus, initially, each display (distractor and target) item receives an equal amount of activation on the output layer.

All other, off-diagonal connections are learnable, with their weights initially set to zero. As these weights project to spatially not corresponding locations, they can be understood to encode shifts of fixations from one location in the input layer to the connected location in the output layer. By disabling changes of the diagonal weights, the network is prevented from learning to increase the weight between a given target location in the input layer and the target location in the output layer, preventing a “trivial” search of the (unique) target element of each display acting as its own “cue” to the target location. Instead, the model is forced to search displays through fixation shifts and thus simulates oculomotor behavior.

The model’s weights are updated at each fixation according to a Hebbian learning rule, including the target fixation. Only the fixation reset to the display center following the fixation on the target is not learned, as it is not part of the search procedure as such. In this way, the weights evolve until the network has searched through the entire set of displays.

An important aspect of the learning is that it takes into account the spatial topography of the search displays. This is achieved by lateral activation of the neurons in the input and output layers (corresponding to currently held and selected next fixation) around their given fixated locations, with the lateral spread decreasing as a function of the distance. Lateral activation is not only physiologically plausible, essentially implementing a kind of population encoding (e.g., Behan & Kime, 1996). Critically, it also enables the network to generalize its behavior to similar, rather than only identical, input patterns: If a saccade from and to similar a region is possible as in a previously executed, spatially similar saccade, the network will be biased towards executing a spatially similar saccade. Specifically, the lateral activations around the current and selected-to-be-next fixation locations are scaled by a negative exponential decay function of the distance to the respective fixation location and, due to the Hebbian learning rule, the change in weights is proportional to the activation between each input and output neuron. Hence, learning can be described by to the following equation:

$$\Delta {w}_{i,j}=\left({e}^{-\varepsilon \left(i,f\left(t\right)\right)/{\tau}_{in}}\right)*\left({e}^{-\varepsilon (j,f(t)/{\tau}_{out}}\right)*lr,$$

whereby

$$\varepsilon \left(k,f\left(l\right)\right) = \sqrt{{\left({x}_{k}-{x}_{f\left(l\right)}\right)}^{2}+{\left({y}_{k}-{y}_{f\left(l\right)}\right)}^{2},}$$

where $\Delta {w}_{i,j}$ is the change of a particular off-diagonal weight between neuron i in the input layer and neuron j in the output layer and $\varepsilon \left(i,f\left(t\right)\right)$ is the Euclidean distance of neuron i to the current fixation location $f\left(t\right)$ in the input layer, while $\varepsilon \left(j,f\left(t+1\right)\right)$ is the distance of neuron j to the selected next fixation location f(t+1) in the output layer. ${\tau}_{in}$ denotes a decay constant which regulates the amount of lateral activation as a function of the distance ε of neuron i from the current fixation location $f\left(t\right)$; analogously, ${\tau}_{out}$ is a decay constant that regulates the amount of lateral activation of neuron j as a function of the distance ε to the selected next fixation location $f\left(t+1\right)$, while lr is the learning rate. Different values of τ correspond to different amounts of spatial generalization/selectivity. The amount of lateral activation in a layer as a function of distance and its dependence on τ is illustrated in Fig. 1D.

In summary, the weight change is proportional to the activations in the input and output layers, with the amount of activation for each neuron scaling according to an exponential decay of distance to the current and future fixation location in the input and output layer, respectively. Critically, while the model must “know” about the identity of fixated target and distractor items (so that it can terminate search), information about the target is not used in any way to induce a, relative to distractors, different form of weight updating in the model—that is, learning is identical for each fixation, whether it falls on a target of a distractor. Hence, the model only learns to associate fixation locations based on consecutive fixation events when searching repeated and nonrepeated arrays.

### Training

The network was initialized 25 times (corresponding to 25 “participants”), every time with a different (randomly generated) set of displays for each individual (simulated) participant. The sample size was based on the minimum effect-size score (of a $\eta {p}^{2}$ = 0.28, 95% CI [0.00, 0.56]) reported in relevant previous studies (e.g., Beesley et al., 2015; Brady & Chun, 2007; Manginelli & Pollmann, 2009; Peterson & Kramer, 2001; Tseng & Li, 2004; Zhao & Ren, 2020). A power analysis conducted with the R (R Core Team, 2023) package *pwr* showed that, to find a context main effect with 85% power, a minimum sample size of *N* = 25 (simulated) participants was required. This sample-size estimation was also comparable with previous (modeling) studies that investigated CC effects using 24 up to 32 simulations (e.g., Brady & Chun, 2007). Based on this estimate, our simulated number of participants (*N* = 25) would be sufficient in terms of its statistical power to detect a possible CC effect with the current model, while also being consistent with previous modeling studies of the CC effect.

Each network underwent 32 training blocks, consisting of 12 repeated/old-context and 12 nonrepeated/new-context trials—in accordance with Chun and Jiang’s (1998) original study and modeling work (Brady & Chun, 2007) on CC. The network was initialized with a ${\tau}_{in}=2.5$ and ${\tau}_{out}=$ 4.5 and a learning rate of 5 × 10–7. The specific values of ${\tau}_{in}$ and ${\tau}_{out}$ were determined empirically, by selecting from simulations according to a previously performed grid-search (e.g., Pontes et al., 2016) across values of ${\tau}_{in}$ and ${\tau}_{out}$, so as to obtain behaviorally plausible values for the number of fixations typically observed in eye-movement studies of CC with stimuli generated analogously to the procedure implemented here (e.g., Manginelli & Pollmann, 2009; Peterson & Kramer, 2001; Zhao & Ren, 2020): Collectively, these studies found that even after a reasonable amount of practice with repeated displays, the number of fixations required by human learners to home in on the target remained relatively high (typically four to six fixations), rather than the target being among the very first items to attract an eye movement. Note that since ${\tau}_{in}$ and ${\tau}_{out}$ are hyperparameters, obtaining an optimal fit by minimizing a cost function with respect to empirical data was neither tractable nor was it the scope of the present work, and ${\tau}_{in}$ and ${\tau}_{out}$ were determined solely according the abovementioned criterion and not regarding the critical observations, detailed in the Results section.

## Results

We investigated whether learning idiosyncratic oculomotor scanning strategies alone would suffice to elicit a CC effect. To this end, we assessed learning by two measures: The first was the number of fixations required to detect the target item, as done in previous work (Beesley et al., 2015; Brady & Chun, 2007). The second measure was inspired by previous work (Seitz et al., 2023) and particularly suited for a network computing oculomotor scan paths, concerned the consistency, or similarity, of the oculomotor scan paths traversed, using Dynamic Time Warping (DTW) computed between each pair of individual repeated-display and, respectively, pairs of nonrepeated-display scan paths (each display containing a unique target position) in each block (for a detailed description, see Seitz et al., 2023), using Python’s similarity-measure library (see Jekel et al., 2019). DTW quantifies the similarity of the shapes of the scan paths with distinct time series by aligning them in the time domain, thus minimizing the Euclidean distance between the aligned series.

To maintain consistency with previous computational work on CC (Beesley et al., 2015; Brady & Chun, 2007) and based on our sample-size estimate, analyses were conducted using significance tests performed on a random sample of participants. Including only a moderate number of participants (25 in the current study, as compared with 32 in Brady & Chun, 2007) avoided artificial inflation of significance values; that is, our study was not over-powered. Importantly, in addition to reporting *p* values, we also report effect sizes ($\eta {p}^{2};$ i.e., the amount of explained variance, which is independent of sample size) as well as confidence intervals.

For the baseline task, we computed repeated-measures ANOVAs with the factors context (repeated, nonrepeated displays) and trial block (1–32)—consistent with the methodology in Seitz et al., (2023, 2024). For comparisons to existing data from the literature (see section titled “Comparison to Empirical Data”), we collapsed eight blocks of trials into one single epoch, yielding a total of four epochs (as the studies considered also analyzed/presented their data across four epochs).

To assess the locality of learning, we computed an ANOVA for the block prior to and after changing distractor elements in the task conditions: that in which the distractor elements in the target quadrant were repositioned (without change in the other quadrants) versus that in which the distractor elements of the quadrant opposite to the target quadrant were repositioned. Finally, for target relocation, an ANOVA was computed for the last block before and after the target was relocated to a position (i.e., swapped position with) a distractor in the opposite quadrant, as well as—for consistency with previous work (Geyer et al., 2024; Manginelli & Pollmann, 2009; Zinchenko et al., 2020)—the epochs (averaging across four trial blocks) before and after target relocation.

### Baseline task: Analysis of oculomotor parameters

**Fig. 2:**
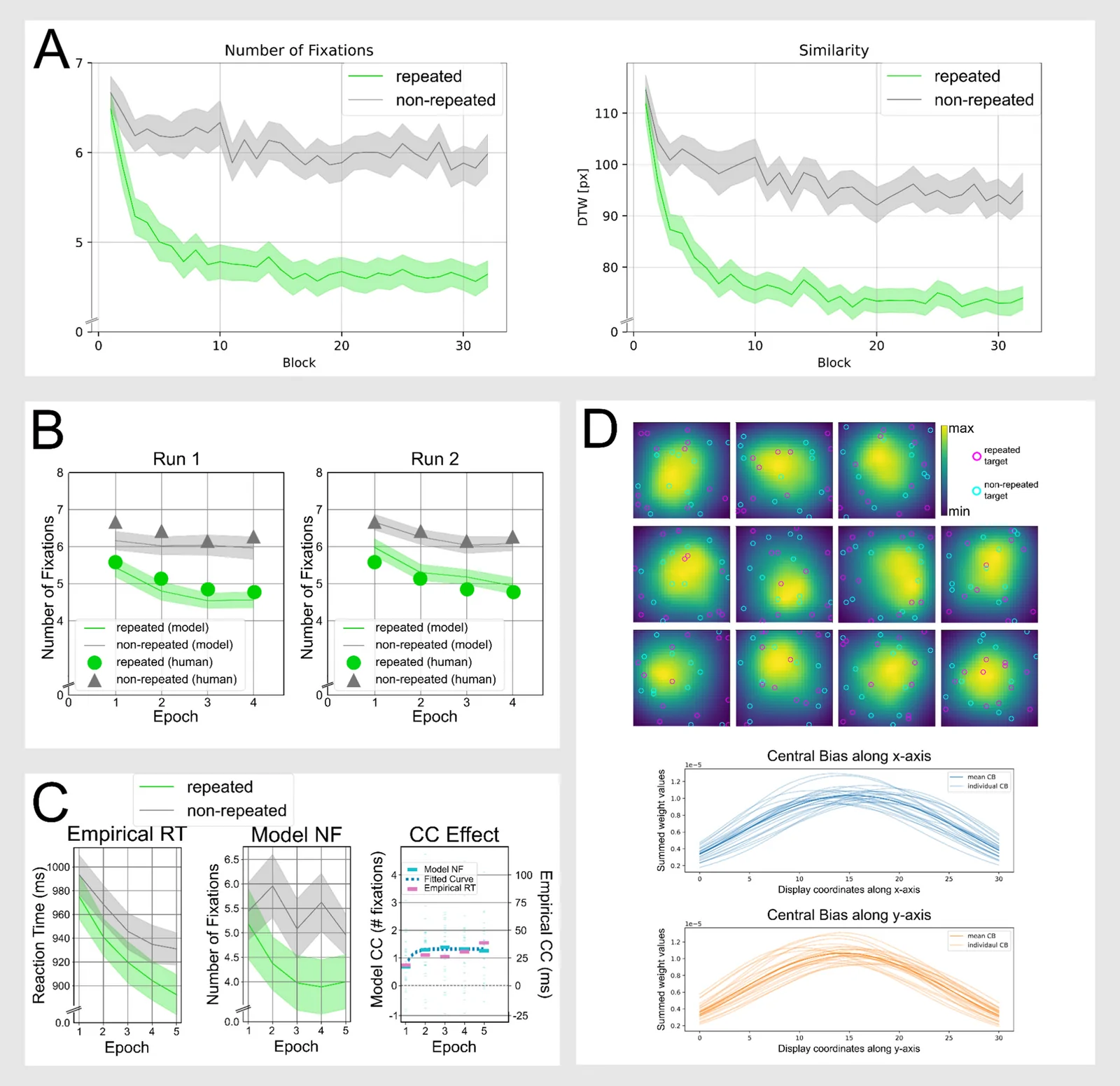
**A** Mean number of fixations (left panel) and the (DTW) similarity (right panel) of the scan paths generated by the network, showing a Block × Context interaction for both measures. **B** Lines show the median number of fixations of the model (“Run 1”), as well as a second, independent version of the experiment (“Run 2”) conducted to validate our findings. One epoch corresponds to > 100 trials. The circular and triangular markers show the median number of fixations from Experiment 1 of Peterson and Kramer (2001). **C** (Left) Empirical RT data from Geyer et al.’s (2024) large sample of participants; (Middle) simulated fixations of our model; (Right) CC effect rendered by the model and human participants (from Geyer et al., 2024), respectively, as well as the curve fitted to the model data, which explains 58% of the variability in both measures. **D** Illustration of the bias of the weights towards the display center, which is an emergent property of our procedural-learning model. The upper panel shows the weights of 11 randomly selected “participants,” while the middle and lower panels show the bias in the *x* and *y* directions, respectively. *Note.* Projections of the fitted 2D Gaussian are not shown because, due to the good fit, they would visually occlude the mean central-bias curves. (Color figure online)

The network showed a significant improvement in the number of fixations required to reach the target for both repeated and nonrepeated displays, main effect of Block, *F*(31, 744) = 19.85, *p* < 0.001, $\eta {p}^{2}$ = 0.45, 95% CI [0.39, 0.49]. Additionally, the network required significantly fewer fixations, on average, to reach the target in repeated versus nonrepeated displays (5.06 vs. 6.18 fixations), main effect of context, *F*(1,24) = 34.32, *p* < 0.001, $\eta {p}^{2}$ = 0.59, 95% CI [0.31, 0.75]. Critically, there was a significant Context × Block interaction, *F*(31,744) = 5.35, *p* < 0.001, $\eta {p}^{2}$ = 0.18, 95% CI [0.11, 0.20] (see Fig. 2A, left panel), reflecting a more marked improvement of performance across blocks for repeated versus nonrepeated displays with increasing practice—evidencing the development of a context, or contextual-facilitation, effect.

Previous research (Seitz et al., 2023) has shown that repeated displays come not only to require fewer fixations, but also that the oculomotor scan paths become more similar as the experiment progresses—more so for repeated than for nonrepeated displays. This effect pattern arises because the oculomotor search strategy itself is being adapted, with repeated displays (by virtue of being encountered repeatedly) weighing in stronger on the optimization of the search strategy than nonrepeated displays (which, by definition, are searched only once; Seitz et al., 2023, 2024). Since our model only learns a procedure—namely, to produce a stereotypical sequence of saccades based on previously executed fixation patterns—we tested whether our network’s scan paths become more similar across blocks and, importantly, whether this effect would be more pronounced for the repeated displays. Indeed, the results revealed Dynamic Time Warping (DTW) scan-path similarity to increase as the experiment progressed, main effect of block: *F*(31,744) = 42.82, *p* < 0.001, $\eta {p}^{2}$ = 0.64, 95% CI [0.60, 0.67], and the scan paths for repeated displays to become more similar than those for nonrepeated displays, main effect of context, *F*(1,24) = 43.82, *p* < 0.001, $\eta {p}^{2}$ = 0.65, 95% CI [0.39, 0.78]. Additionally, there was a significant Context × Block interaction, *F*(31,744) = 4.39, *p* < 0.001, $\eta {p}^{2}$ = 0.15, 95% CI [0.09, 0.17] (Fig. 2A, right panel), evidencing a more marked increase in scanpath similarity across blocks for repeated versus nonrepeated displays. We obtained the same result pattern in a second run (see Fig. 2B) for which we generated a new set of nonrepeated/repeated displays—thus rendering it unlikely that the first set of results was driven by specific properties of the displays in the first run. This pattern mirrors the human data.

### Comparison to empirical data

We went on to examine how well our model fits with empirical data. For repeated displays, the median number of fixations required to reach the target was above 4, while it was above 5 for nonrepeated displays. These numbers match previous reports well, including Peterson and Kramer’s (2001) benchmark oculomotor study of CC. Figure 2B gives the median number of fixations of the simulation as well as that reported by Peterson and Kramer (2001). To assess the fit quantitatively, we computed the Mean Absolute Error between the model’s results and Peterson and Kramer’s data for both runs, which was 0.3 fixations (largest deviation = 0.5, relative error = 5%) for Run 1 and 0.2 fixations (largest deviation = 0.6, relative error = 3%) for Run 2.

Next, we fitted a curve to the contextual-facilitation effect generated by the model (in accordance with the approach taken by Brady & Chun, 2007), which was best described by a power function (see Chun & Jiang, 2003). The variance explained was 98%, indicating that the function provides a good fit of the model-produced values. Subsequently, we calculated the model-produced CC effect, measured in terms of (the savings of) fixation number in repeated versus nonrepeated displays, and that from real human participants’ (Geyer et al., 2024), measured in (nonrepeated minus repeated) reaction times, in a common metric space (Fig. 2C)—based on the premise that a single fixation’s savings correspond to a 25-ms gain in processing time (e.g., Wolfe, 1994). We then fitted the two dependent variables (the model-produced and human-produced CC effects) with a single two-parameter power function relating the CC effect to time on task in terms of experimental epochs (with one epoch summarizing > 100 trials). This yielded the magnitude of the CC effect, y, per epoch, x, as follows:

$$y=16.30*\left(1-{e}^{\left(-\frac{x}{0.32}\right)}\right)-14.97.$$

This power function explained 58% of the variance in both dependent variables. Assuming that contextual facilitation approaches an asymptotic value across epochs is also more plausible compared to a (monotonically increasing) logarithmic relationship (Brady & Chun, 2007). Fixation numbers and reaction times have a natural lower bound (i.e., ≥0) and their characteristics over time are best described by power functions (e.g., Anderson, 1982; Chun & Jiang, 2003; Fitts & Posner, 1967). Consequently, rather than growing unboundedly, the difference between increasingly smaller values must approach some asymptotic value. Figure 2D illustrates our findings.

### Central bias as emergent phenomenon of procedural learning

Finally, we examined where our models “looks” after training, by summing up the learnt weights for individual (output) locations across displays and “participants.” One might have expected the network to display a strong bias towards individual target locations. Somewhat surprisingly, however, it actually developed a bias to scan particularly central display locations (not necessarily coinciding with target positions)—an emergent behavior mimicking human visual exploration (e.g., Clarke & Tatler, 2014). This is illustrated in Fig. 2D, which depicts the summed weights of randomly selected “participants.” As can be seen, the weights do not project in a 1:1 fashion to single target locations in repeated and nonrepeated displays; instead, they cluster around the central display region. Such a bias was also seen to materialize in other (control) simulations, in which we had the model start search at some peripheral (rather than the central) location on each trial (e.g., the location in the top-left display quadrant, coordinates: x=3,y=3 in the 31 × 31 matrix). It is, thus, unlikely that the central bias is a particular consequence of our training procedure, with search, by default, starting at the display center (coordinates: x=15,y=15). Rather, the central bias is likely to reflect training-induced plasticity in the process of visual search akin to human behavior (providing an alternative account on the observations by Tatler, 2007).

In support of this, fitting a bivariate Gaussian (with a least-squares approximation) to the average bias of all “participants,” along the lines of Clarke and Tatler (2014), we found the computational data to be well described by a truncated Gaussian, with the center of the Gaussian located at the display-center coordinates x=15.4, y=14.5, with a standard deviation of 10.5 and 10.1 in the x and y directions, respectively. To assess the goodness of fit, we computed the mean average error between the bias values and the fitted Gaussian, divided by the mean bias value, which yielded a relative error of 1.7%.

### Behavior relating to theoretical predictions: Locality of cueing and relocation cost

Finally, we assessed the model’s behavior, in terms of the number of required fixations, regarding two theory-derived predictions, namely: the cueing effect is stronger (for distractors) in the vicinity of the target (Olson & Chun, 2002; Brady & Chun, 2007), and relocating the target (to the opposite to the original display quadrant) in repeated displays comes with a cost (Manginelli & Pollmann, 2009; Zinchenko et al., 2020).

With changes of the positions of distractors in the target quadrant (from baseline Block 32 to experimental Block 33), a large cueing effect prior to the change turned into a small, but persistent, effect after the change. Statistically, there was a main effect of context, *F*(1,24) = 34.94, *p* < 0.001, $\eta {p}^{2}$ = 0.59, 95% CI [0.31, 0.75], along with a significant main effect of block, *F*(1,24) = 32.76, *p* < 0.001, $\eta {p}^{2}$ = 0.58, 95% CI [0.29, 0.74], the latter reflecting the cost of change. Critically, the Context × Block interaction was significant, *F*(1,24) = 13.96, *p* = 0.001, $\eta {p}^{2}$ = 0.37, 95% CI [0.08, 0.59], attesting to the cost effect: The number of fixations required to select the target in repeated displays was larger after compared with before changing the distractor positions (but not the target position) within the target quadrant, *t*(24) = 8.81,* p* < 0.001. In contrast, no cost effect was evident when changing the positions of the distractors in the quadrant opposite to the target (while leaving those in the target quadrant unchanged). An ANOVA of the required number of fixations revealed only a main effect of context, *F*(1,24) = 30.35, *p* < 0.001, $\eta {p}^{2}$ = 0.56, 95% CI [0.27, 0.73], indicative of overall contextual facilitation. But neither the main effect of block, *F*(1,24) = 0.90, *p* = 0.35, $\eta {p}^{2}$ = 0.04, 95% CI [0.00, 0.26]) nor the Context × Block interaction, *F*(1,24) = 0.16, *p* = 0.69, $\eta {p}^{2}$ = 0.00, 95% CI [0.00, 0.18], were significant, and a post hoc *t* test of the required number or fixations in repeated displays before and after the change was indicative of the absence of a difference, *t*(24) = − 0.57, *p* = 0.58, with the associated Bayes-factor, BF = 1.4 (not favoring the hypothesis of a cost).

The other prediction we tested concerned the effect of target relocation. Here, we carried out two ANOVAs, one for the number of fixations in the block before and after relocation, and one for the “epoch” (each epoch averaging across four trials) before and after relocation. The latter analysis ensured comparability with the previous literature (Brady & Chun, 2007; Chun & Jiang, 1998; Geyer et al., 2024; Manginelli & Pollmann, 2009; Peterson & Kramer, 2001; Zinchenko et al., 2020), which, in these studies, was aimed at increasing statistical power. On the level of blocks, the main effect of context was nonsignificant or only marginally significant, *F*(1,24) = 2.57, *p* = 0.056, $\eta {p}^{2}$ = 0.10, 95% CI [0.00, 0.35], indicative of a marginal overall-facilitation effect. Critically, both the main effect of block, *F*(1,24) = 37.87, *p* < 0.001, $\eta {p}^{2}$ = 0.61, 95% CI [0.34, 0.76]) and the Context × Block interaction, *F*(1,24) = 35.93, *p* < 0.001, $\eta {p}^{2}$ = 0.60, 95% CI [0.32, 0.75], were significant: The facilitation effect was reduced in the block after compared to that before the target relocation, owing to an increase in the number of fixations required to select the target in repeated displays—the relocation cost. The results were essentially similar for the analysis on the level of epochs, except the main effect of context remained significant, *F*(1,24) = 8.48, *p* = 0.008, $\eta {p}^{2}$ = 0.26, 95% CI [0.02, 0.51]); epoch: *F*(1,24) = 28.73, *p* < 0.001, $\eta {p}^{2}$ = 0.54, 95% CI [0.25, 0.72]; Context × Epoch interaction, *F*(1,24) = 24.43, *p* < 0.001, $\eta {p}^{2}$ = 0.50, 95% CI [0.21, 0.69]. This is consistent with a gradual recovery of cueing in the relocation epoch (Geyer et al., 2024; Manginelli & Pollmann, 2009), as attested by the smaller $\eta {p}^{2}$ = 0.26 compared with $\eta {p}^{2}$ = 0.59 for the epoch before relocation.

## Discussion

The present study demonstrates that acquiring a display-general oculomotor scanning procedure in a visual search task can yield a CC effect even without learning display-to-target mappings of the type assumed by previous contextual-memory models. Our model learns a distributed transition policy whose elementary units are local fixation-to-fixation associations rather than display-to-target mappings: If a saccade from a given fixated region to another (among several alternatives) is possible and the model has previously executed a similar saccade, the model is biased to execute that saccade over other possible ones. While this limits performance, in that nontarget elements will inevitably also be visited during search, the saccades ultimately terminate at the task-critical target locations. The network thus indirectly learns to generalize its search behavior in such a way that many saccades are biased towards target locations—in line with previous computational models of the CC effect (Beesley et al., 2015; Brady & Chun, 2007). Critically, the learning signal is defined over sequential fixation transitions, so that the model acquires a policy over scan-path trajectories rather than a mapping from stimulus configurations to target locations. This perspective is closely related to the notion of habitual attention (Y. V. Jiang, 2018), which proposes that repeated experience can shape how attention is sequentially allocated through a scene, rather than forming explicit target-location representations (“learning that”). From this viewpoint, contextual learning can be understood as the acquisition of attentional routines (“learning how”), consistent with the procedural scan-path learning mechanism implemented here. Importantly, although our model operates on a spatial input representation of the display, learning is not driven by a mapping from full stimulus configurations to target locations. We acknowledge, however, that because every search episode terminates with a fixation on the target, the learning rule inevitably reinforces local transitions into the target. Importantly, the target is special because it terminates search, not because it is treated as a privileged teaching signal by the learning rule. Computationally, it constitutes the terminal state of the same transition-learning mechanism that links every pair of consecutive fixations. Instead, weight updates are contingent on the currently executed fixation and the immediate transition to the next selected location. As a consequence, only locally visited states contribute to learning, and unvisited stimulus elements are not directly associated with target identity or target location. This implies that the model does not implement a display-to-target associative lookup, but rather learns a policy over sequential state transitions between fixated locations. While stimulus configurations influence the available transitions at each step, they do not serve as supervised input patterns that are directly mapped onto target locations.

Worth mentioning is that our learning scheme also differs from standard reinforcement-learning accounts in which the outcome of a whole trial is used to adjust earlier steps in the sequence. In such accounts, successful completion of a search episode can lead to earlier actions being strengthened more strongly than later ones, because the system “assigns credit” backward through the sequence of choices that led to success. Our model does not operate in this way. Instead, learning occurs locally and immediately at each eye movement. Each saccade is reinforced only in relation to the next saccade that follows it, independent of whether the overall trial ends successfully or where in the sequence the saccade occurs. This means that the fact that every trial ends once the target is found does not change how learning is distributed across the sequence. There is no special strengthening of the final eye movement, and no mechanism that spreads information about success backward to earlier steps. As a result, all parts of a scan path are treated equally in learning terms: What matters is only the local transition from one fixation to the next, not whether that transition happened early or late in a trial.

An important implication of this distinction is that display-to-target associations alone are unlikely to account for the contextual-cueing effect produced by the model. We acknowledge that every search episode terminates with a transition into the target and that this transition is therefore reinforced whenever it is executed. However, this final transition represents only one element within a much longer sequence of fixation transitions. Every search episode contains numerous distractor-to-distractor transitions, whereas only a single transition terminates at the target. Consequently, learning is distributed across the entire scan path rather than being concentrated on the final eye movement. This interpretation is also consistent with the behavioral measures considered in the present study. Beyond reproducing the reduction in the number of fixations required to locate the target, the model also reproduces the increasing similarity of complete scan paths, quantified by DTW. Because DTW captures the similarity of entire fixation sequences rather than isolated eye movements, the observed increase in scan-path similarity is difficult to reconcile with an account based primarily on reinforcement of a final distractor-to-target transition alone. Instead, it suggests that repeated experience gradually shapes the complete oculomotor trajectory. Accordingly, while local distractor-to-target associations naturally emerge as part of the learning process, which is supported by empirical evidence, we argue that they represent only one component of a distributed procedural policy over fixation transitions, rather than the display-specific display-to-target associations assumed by previous contextual-memory models.

While our results demonstrate that procedurally learnt, display-general oculomotor scanning routines go a long way to account for CC, they do not rule out other mechanisms—of top-down search “guidance”—proposed in the CC literature: these assume associative learning of specific, repeatedly encountered spatial distractor–target relations (LTM “templates”), which, when activated by a given repeated display, bias the deployment of attention towards the target location (e.g., Wolfe, 2020), possibly augmented by facilitated response-selection and/or response-execution processes (e.g., Kunar et al., 2007). In this sense, we acknowledge that improvements in task performance over time-on-task may also arise from processes not directly related to visual search guidance. For example, participants may become more efficient in issuing speeded motor responses, improve discrimination of target from distractor shapes, or gain familiarity with the temporal structure of the task. Our aim is not to exhaustively decompose these sources of learning, but to demonstrate that a purely procedural mechanism operating on oculomotor transition structure is sufficient to account for key signatures of contextual cueing, including reduced numbers of fixations for repeated compared to nonrepeated displays and systematic changes in scan-path organization.

Evidence for display-specific contextual cueing comes from, for example, electrophysiological studies that, critically, disallowed eye movements to minimize EEG artifacts (e.g., Johnson et al., 2007; Schankin & Schubö, 2009; Stokes et al., 2012; Zinchenko et al., 2020). Examining lateralized event-related potential (ERP) components, such as the N2pc (e.g., Luck, 2014), to track the (lateralized) allocation of covert attention (e.g., Liesefeld et al., 2017), their results indicate that focal attention may be deployed directly to the target location in individual repeated displays. Moreover, models like Brady and Chun’s (2007) or Beesley et al. (2015) could successfully recreate CC effects when considering individual (specific) displays and the learning of individual target or distractor locations in these displays. Recall that in CC experiments, typically 8–12 old-context displays of different spatial compositions are repeatedly presented (for review, see, e.g., Goujon et al., 2015). Given this limited number, the previous models might, in a certain sense, also be considered as “display-general”—in that weights are established such that they can accommodate the full range of input patterns across the entire set of (8–12) repeated arrays. However, what distinguishes these models from the current model is how the weight updating is achieved. While the existing models (Beesley et al., 2015; Brady & Chum, 2007) update weights for individual display items that appear at particular *x/y* input locations, our model iteratively updates weights in an entire region around the currently fixated item, irrespective of the identity—target or distractor—of that item. Thus, weights are established in a spatiotopic (i.e., screen-centered) format and relatively independently of the specific *x/y* coordinates of the items in individual displays. As a consequence, learned input–output linkages can be also activated from items in other search arrays. Accordingly, we use the terms display-specific and display-general to describe how practice-related performance improvements are brought about by the respective models with time on task. To reiterate: Empirical evidence and theoretical considerations suggest that contextual facilitation of visual search can be attributed to the build-up of (a set of) display-specific LTM representations about specific (repeated) display arrangements that, on later occasions, guide search to the target location more effectively than arrangements not represented in LTM (e.g., Beesley et al., 2015; Logan, 1988; Brady & Chun, 2007), by activation of the LTM representation raising the selection priority of the target location. However, other evidence, empirical (e.g., Seitz et al., 2023) as well as theoretical (from the present, modeling, study), also supports process-based accounts of contextual learning, according to which routinization of the process of visual search (i.e., the “proceduralization” of oculomotor scanning) as such can lead to optimal levels of performance (cf. Shiffrin & Schneider’s, 1977, notion of “automaticity”). Ultimately, the empirical question is under which conditions contextual facilitation is (more) display-specific or (more) display-general. We conjecture that disallowing eye movements fosters display-specific learning. Accordingly, in the electrophysiological studies mentioned above, an individual distractor layout may come to “cue” attention to an individual (specific) target position. However, the task demands in the electrophysiological studies are quite different to those under more “naturalistic” conditions which allow eye movements, including virtual-reality environments (e.g., Marek & Pollmann, 2020; Sonkusare et al., 2019). By allowing oculomotor scanning, such scenarios foster display-general learning in the form of the development of effective scanning routines adapted to (optimized for) the set of search displays repeatedly encountered. This is an issue for future research.

In fact, there already is empirical evidence of display-specific learning coexisting with procedural learning (Seitz et al., 2024; Zinchenko et al., 2026). Further, like most theoretical accounts of CC, the current model is based on searching artificial letter arrays—where it is not clear to what extent the cueing effect involves explicit display recognition (e.g., see Chun, 2000; Chun & Jiang, 1998; Colagiuri & Livesey, 2016; Y. Jiang & Chun, 2003; Vadillo et al., 2016; Kroell et al., 2019; Meyen et al., 2023). For naturalistic images, by contrast, explicit recognition is typically observed (see Brockmole & Henderson, 2006), along with eye movements instantly approaching the target (Summerfield et al., 2006). In the extant models of CC in artificial arrays (Beesley et al., 2015; Brady & Chun, 2007), learning is constrained around the target location and, one a display has been sufficiently learnt, the “readout” of search-guiding information in the output layer occurs instantaneously. Hence, for a given learnt display, the acquired weights between the distractor items and the target location will activate the target’s position sufficiently to be likely the first location checked. Note that even in small (8 × 6) displays, the statistically occurring overlap among the various item configurations is low, especially as regards the items surrounding the target which have a greater influence. As a result, each display is likely to activate only one target location, with little, if any, interference from other displays—providing an additional, mathematical argument for inherently display-specific (rather than display-generic) contextual learning in the previous models (Beesley et al., 2015; Brady & Chun, 2007). Given this, we conjecture that these models are better applicable to scenarios allowing instantaneous (explicit) recognition of learnt displays (akin to in naturalistic scenarios), whereas the approach developed here is better suited to for scenarios that do not support immediate recognition, in which case the scanning procedure becomes more relevant.

Of note, our network does not claim detailed neurobiological plausibility; for instance, it does not endeavor to capture the cellular operation of neurons in the superior colliculus (e.g., Van Opstal & Van Gisbergen, 1990). Rather, it was designed to provide a computationally explicit, in-principle implementation of a hypothesis according to which the mere acquisition of a simple, “mindless” procedure suffices to generate contextual facilitation—thus, questioning the theoretical necessity to assume (learnt) display-specific LTM representations to account for CC effects (without, of course, ruling out such representations). Of importance in this regard, our model produces a reasonable fit to empirical data obtained from human participants: even at the end of learning, the number of fixations required to find the target remains above 4 (or 5) for repeated (and, respectively, nonrepeated) displays (consistent with the reports of Peterson & Kramer, 2001; Tseng & Li, 2004)—rather than the empirically implausible one or two fixations estimated by Brady and Chun’s (2007) model for repeated displays.

This puts our model of procedural context learning on a similar level to that proposed by, for example, Itti and Koch (2001) for salience and the published models of CC (Beesley et al., 2015; Brady & Chun, 2007). The central aim of our endeavor was to develop a computationally explicit hypothesis (i.e., a new type of model) based on a theoretical motivation derived from empirical data, rather than to optimize the fit between an (already established) model and empirical data. Here, the proposed model is that CC results from an associative learning between fixational regions, based on prior history of salience.

Beyond the central finding of contextual facilitation from learning a procedure, we observed a central bias as an emergent property of procedural learning. While it is thought that a central bias can arise from training with specific target locations (Parkhurst et al., 2002), our model suggests that just performing “mindless” scanning of the visual displays may suffice for the bias to emerge—in line with the observation of a central bias even in (target-less) free-viewing tasks (Carnosa et al., 2003). Computationally, this is a consequence of associating regions within a constrained frame, by which central proportions of the display accrue more weight over time. As this is the result of lateral activations, as it can also be observed in biological systems (Behan & Kime, 1996), the central bias observed in human participants may have a similar origin.

Crucially, here we provide a procedural account of learning that can render performance improvements in visual search that are not involve the learning of specific target locations. Our modeling results support the (procedural) learning of oculomotor trajectories to be a potentially important factor that has so far not been explicitly considered in models of visual search, such as the prominent Guided Search (e.g., Wolfe, 2021). On this account, routinized eye movements, rather than being the mere consequence of “targeted” shifts of attention, promote the orienting of attention toward display regions that (based on oculomotor-sampling experience) most likely contain relevant information for attentional processing—prior to the selection of specific locations/items based on bottom-up or top-down “cues.” Accordingly, we propose that procedural learning plays an active role in visual search in the sense that convenient, general search routines optimally adapted to the “world” of displays encountered constitute another factor—besides the allocation of attention based on acquired target-distractor LT memories—that aid the allocation of attention in a manner unguided by other feature-based (i.e., bottom-up or more short-term top-down) factors.

Arguably, this type of learning is applicable to skill acquisition in general, which constitutes a universal characteristic of human behavior (Fitts, 1954; Fitts & Poser, 1967; Luchins, 1942; Ryle, 1946; Willingham et al., 1989). Although here we explore its potential with regard to a particular phenomenon—contextual facilitation—we believe that the notion of skill acquisition has implications for a model-based understanding of oculomotor behavior in visual exploration and scene viewing in general.

### Summary and outlook

The key finding from our modeling work is that a network employing solely procedural learning can give rise to what looks like memory-based “cueing” of attention to the target location in a hard visual-search task. Importantly, the model is never informed about the identity of individual items, nor does it learn to associate a target location with the distractor layout. Hence, it does not acquire any “knowing that” (Ryle, 1946). Rather, it just learns to adapt, and hone, its previously executed fixation patterns, with optimization occurring incidentally, simply as a byproduct of repeatedly searching a certain set of displays. The network thus acquires “knowing how” to best solve the task. Although here a motor procedure is learned, we suggest that the findings generalize to other forms of procedural learning.

While the model does not acquire specific context-target associations (“templates” in long-term memory) that top-down guide attentional selection (contextual “cueing”), it nevertheless facilitates search by learning a display-generic oculomotor procedure. This raises the question whether the findings from other statistical learning paradigms (e.g., Bogaerts et al., 2022; Theeuwes et al., 2022) may also be explained by the acquisition of (oculo-)motor routines (i.e., “learning how”), rather than preattentive guidance processes (i.e., “learning that”). We hypothesize that if a simple procedure can be readily set up to solve the task at hand (e.g., visual search), attentional guidance may actually not be the dominant mechanism driving the learning-dependent performance gains. Accordingly, future research would need to factor in participants’ capacity for procedural learning across a range of cognitive domains and tasks, which would require innovative experimental designs to separate procedural and attentional-guidance effects and/or establish to what extent procedural learning interacts with statistical learning of attentional guidance. Leveraging scenarios in which acquired procedures render performance errors whereas “cognitive” strategies would yield the correct outcome might be particularly insightful in investigating the interaction between “learning how” and “learning what” in an adaptive manner.

With regard to conceptual and computational models of (foveated) vision that have traditionally emphasized guidance by bottom-up and/or top-down information: we argue that, in light of our empirical and modeling results, these models might profitably consider whether procedural strategies may not equally well explain existing findings, or in what way the models may be augmented by incorporating procedural learning. Cases in point would be considering whether the central bias is an emergent property of a statistical “learning-how” mechanism, or whether architectures operating on selecting “glimpses” (e.g., Mnih et al., 2014) would benefit from procedurally learnt stable fixation policies. Of particular theoretical, as well as practical, interest is that, in our model, learning a simple procedure rendered an advantage in finding the target without any explicit “teaching signal” informing the network about target identity or location. This finding may hold potential for the domain of machine learning and artificial intelligence—namely, by employing learning strategies requiring less supervision by (e.g., developing loss functions or training schemes) focusing on “learning how” instead of “learning that”—directly leveraging the task structure itself rather than the outcome of single trials. Generally, we believe it would be profitable tested whether some tasks, particularly involving statistical learning, may be explained and successfully modelled as instances of procedural learning.

## Data Availability

Not applicable—we did not collect data from human participants for this study.

## Footnotes

1 We evoke a view of optimization according to which visual search is adjusted toward a specific goal—namely, (in our paradigm) finding and responding to a target letter “T” in a cluttered array of distractor letters “L.” Given that this target differs from the distractors only in the combination of two shape features and the “T” versus “L” junction, the search as such is likely “inefficient,” characterized by relatively steep slopes of the function relating RT to the number of items in the search display (e.g., Wolfe, 2021) and/or ineffective oculomotor patterns. Nevertheless, through procedural learning, performance is optimized to achieve the goal reliably with a minimum of effort.

## Code availability

The code will be made available upon request.
